# Effect of Biomass Fuel Use on Neonatal Outcomes: A Cohort Study of Pregnant Females

**DOI:** 10.3390/ijerph22091336

**Published:** 2025-08-27

**Authors:** Rajitha Wickremasinghe, Meghan Tipre, Ruwanthi Perera, Pavithra Godamunne, Rodney R. Larson, Mark Leader, Claudiu T. Lungu, Udaya Wimalasiri, Priyantha Perera, Sumal Nandasena

**Affiliations:** 1 Department of Public Health, Faculty of Medicine, University of Kelaniya, P.O. Box 6, Thalagolla Road, Ragama 11010, Sri Lanka; udayaforphd@gmail.com; 2 Division of Haematology and Oncology, School of Medicine, The University of Pittsburgh, Pittsburgh, PA 15261, USA; met169@pitt.edu; 3 Department of Rogavijnana, Faculty of Indigenous Medicine, Gampaha Wickramarachchi University of Indigenous Medicine, Yakkala 11870, Sri Lanka; bprperera@gmail.com; 4 Department of Medical Education, Faculty of Medicine, University of Kelaniya, P.O. Box 6, Thalagolla Road, Ragama 11010, Sri Lanka; pavithrag@kln.ac.lk; 5 Larson International, LLC, 1656 Longwood Glen Lane, Friendswood, TX 77546, USA; rlarsoncih@aol.com; 6 Department of Epidemiology, School of Public Health, University of Alabama at Birmingham, Birmingham, AL 35294, USA; markdleader@gmail.com; 7 Department of Environmental Health Sciences, School of Public Health, University of Alabama at Birmingham, Birmingham, AL 35294, USA; clungu@uab.edu; 8 Department of Paediatrics, Faculty of Medicine, University of Kelaniya, P.O. Box 6, Thalagolla Road, Ragama 11010, Sri Lanka; priyanthap@wyb.ac.lk; 9 Provincial Director of Health Office Western Province, Maligawatte Secretariat Building, P.O. Box 876, Colombo 00100, Sri Lanka; sumalnandasena@gmail.com

**Keywords:** indoor air pollution, pregnancy outcomes, biomass combustion, low birth weight

## Abstract

**Background:** Exposure to indoor air pollution (IAP), including particulate matter of size 2.5 µm/m^3^ (PM_2.5_) and carbon monoxide (CO) resulting from the combustion of biomass fuels in homes, is an important risk factor associated with growth and developmental delays in neonates. We investigated the association between exposure to HAP and adverse birth outcomes in a birth cohort study of 594 pregnant females in Sri Lanka. **Methods**: Pregnant females between the ages of 18 and 40 years were enrolled in their first trimester and followed until delivery. Baseline assessments of fuel used for cooking were used to categorize the females into high-exposure (wood and kerosene) or low-exposure (liquid petroleum gas and electricity) groups. Indoor air quality measurements of PM_2.5_ (n = 303) and CO (n = 258) were conducted in a subgroup of households. The outcomes at birth included the neonates’ appearance, pulse, grimace, activity, respiration (APGAR) score, Brazelton Neonatal Behavioural Assessment Scale (BNBAS) score, and birth weight. Linear and logistic regressions were used to evaluate the association between household air pollution (HAP) and birth outcomes. **Results**: Of the 526 neonates assessed at delivery, 55.7% were born to mothers with high HAP exposure and 44.3% with low HAP exposure, respectively. The results of the linear regression found an inverse association between higher exposure to HAP and birthweight in the adjusted and unadjusted models; the birth weight of children in the high-exposure group was lower by 107 g compared to that of the low-exposure group after adjusting for other variables (β = −106.8; 95% confidence intervals: −197.6, −16.0). Exposure status was not associated with birth length, gestational age, or the APGAR score; however, the BNBAS motor score was significantly lower in the neonates of the high-exposure group (6.41 vs. 6.55, *p* = 0.04), though it was not significant when adjusted for other variables. No correlation was found between the measured indoor PM_2.5_ levels and birth weight, birth length, gestational age, APGAR score, or BNBAS score. **Conclusions**: Exposure to IAP due to emissions from combustion products from biomass fuels adversely affects birth weight. These effects may be more pronounced in vulnerable populations in settings where primary healthcare for pregnant women is limited.

## 1. Introduction

Indoor air quality (IAQ) is an important environmental factor that affects health, especially in vulnerable groups like children, the elderly, and those who suffer from chronic respiratory and/or cardiovascular diseases [1]. It has been estimated that people spend about 90% of their time in indoor environments, which include homes, workplaces, schools, etc. Hence, for many people the indoor air quality may have a greater impact on their health than the outdoor air quality [2].

Exposure to higher levels of indoor air pollution (IAP) during pregnancy may have adverse effects on pregnancy outcomes, birth outcomes, and beyond into childhood and adult life. A meta-analysis that evaluated the association between exposure to IAP from biomass fuel and low birth weight in sub-Saharan Africa found a 74% increased risk of having a low-birth-weight baby among mothers exposed to higher levels of IAP [3]. The rate of physical growth during infancy is at its maximum. During the first year of life, the weight increases by 200% and the length by 50% compared to the parameters at birth, which may be affected by exposure to IAP during pregnancy [4]. Growth is a complex interaction between genetics and the environment. Exposure to toxins is a known risk factor for limiting or otherwise affecting growth and development.

Several studies have investigated the association between maternal exposure to IAP and adverse birth outcomes, such as low birth weight and small-for-gestational-age; however, in many of those studies, tobacco smoking and/or exposure to passive smoking was a strong confounding factor [5,6,7,8,9]. Oftentimes, these studies relied on self-reported exposure to IAP and lacked air quality measurements. A previous study conducted among rural communities in the Central Province of Sri Lanka investigated the association between exposure to air pollution caused by emissions from the combustion of biomass fuels and adverse foetal growth outcomes; exposure increased the risk of low birth weight (adjusted odds ratio (aOR) = 2.74) and being small-for-gestational-age (aOR = 1.87) [10].

IAP is affected primarily by three factors: outdoor air quality, human activity in buildings, and building construction materials [1]. Among them, human activities, such as the burning of household waste, tobacco smoke, pesticides and solvents, cleaning agents, combustion sources, and cooking activity, contribute to a reduction in air quality. Combustion sources and cooking activity are particularly important in low- and middle-income country settings. In Sri Lanka, an island country in the Indian sub-continent, nearly 66% of the population uses biomass fuel for indoor cooking [11]. It was estimated that IAP is a larger threat to health than outdoor air pollution. Although more than 80% of Sri Lankan households have electricity, it is used primarily for lighting, and biomass (usually wood) is used for cooking [12]. A study of the profile of biomass stoves used in Sri Lanka reported that biomass stove emissions influence Sri Lanka’s IAP more than any other factor; the emissions from traditional biomass fuel stoves include carbon monoxide (CO), nitrous oxide, sulphur oxides, and other organic compounds and, more importantly, particulate matter (PM_2.5_), which penetrates into the alveolar region located deep in the lungs. As most of the cooking is performed by women, who are accompanied by their young children and possibly elderly family members present in the cooking area, they are the groups vulnerable to the harmful effects associated with exposure to these contaminants.

A study conducted on children’s environmental health revealed that the majority of under-five deaths in Sri Lanka are neonatal; the evidence to support an environmental association is lacking [13]. Only a limited number of epidemiological studies in Sri Lanka have investigated the health effects of air pollution [14], and only one study has investigated the association between maternal exposure to IAP and adverse birth outcomes [10].

To address some of the gaps in the literature and to add to the body of existing literature, investigators from the University of Kelaniya (UKe) in Sri Lanka and the University of Alabama at Birmingham, United States, conducted a birth cohort study in Sri Lanka to investigate the effects of prenatal and early childhood exposure to IAP on birth outcomes, infant neurodevelopment, and early child development. In this paper, we examine the relationship between prenatal exposure to specific emissions from the combustion of biomass fuels and adverse birth outcomes.

## 2. Materials and Methods

### 2.1. Design and Setting

A prospective design was used to assemble a cohort of pregnant females in the Ragama Medical Officer of Health (MOH) area in the Gampaha district of Sri Lanka. The Gampaha district is the second most populous district of the country, with a 2.3 million population residing in a 1589 square kilometres area [15]. The district has urban, semi-urban, and rural populations and is diverse ethnically. The Ragama MOH area is the field practice area for the Faculty of Medicine, University of Kelaniya (UKe), a state university in Sri Lanka. The study area is divided into 13 public health midwife (PHM) areas. Almost 90% of pregnant women register at field antenatal clinics before the 12th week of gestation. Pregnant females needing specialized care are referred to the antenatal clinics at the Colombo North Teaching Hospital (CNTH), Ragama, the teaching hospital of the Faculty of Medicine, UKe. The recruitment of pregnant females who were eligible was carried out in the following sites: (1) field antenatal clinics in the Ragama MOH area and (2) antenatal clinics conducted at the CNTH.

### 2.2. Study Population

The study population comprised pregnant females in their first trimester residing in the Ragama MOH area and who visited the antenatal clinics as mentioned above. The inclusion criteria were women who (1) were permanent residents in the Ragama MOH area; (2) were between 18 and 40 years of age; (3) were <12 weeks pregnant (1st trimester of pregnancy) at the time of enrolment; (4) planned to continue antenatal care and deliver at the CNTH; (4) had a singleton birth; and (5) consented to participate in the study. The exclusion criteria were mothers with diabetes, hypertension, multiple pregnancies, or who delivered a baby with a major congenital anomaly. We also excluded mothers who claimed that they were not involved in cooking routinely or were full-time working mothers.

### 2.3. Recruitment of Study Participants

The research staff at the UKe worked closely with the public health staff of the Ragama MOH area (i.e., primary health care workers, including PHMs and medical officers of health) to identify eligible women. The pregnant mothers’ registry maintained by the PHM was used as the sampling frame. The PHM is the female grass-roots level family health worker primarily responsible for maternal and child health; the Public Health Inspector (PHI) is responsible for environmental health, communicable disease prevention, and food safety. The PHM is responsible for registering all eligible couples (a married couple with the female partner within the child-bearing ages of 15 to 49 years) and children under five in her assigned area, and maintaining the respective registers. The PHM is expected to visit each pregnant female’s home twice during the first 10 days after delivery and then on a regular basis. At delivery, a Child Health Development Record (CHDR) is issued to each child by the hospital, which helps to monitor the child’s growth until 18 years of age. The CHDR consists of information from birth, including immunization; serially monitored weight and length/height from birth; and notes written by the MOH, the PHM, and also by other treating physicians for special conditions. The PHM maintains a duplicate copy of the CHDR with essential information. The RAs identified eligible pregnant females, explained the study to them in detail, and obtained their written consent to participate. Recruitment commenced in July 2011. All data collection procedures were completed in June 2014.

### 2.4. Data Collection Procedures

#### 2.4.1. Baseline Questionnaire

At enrolment, the Research Assistants (RAs) administered a baseline questionnaire which ascertained information on socio-demographic characteristics, including age, education, occupation of mother and father, income and assets, and number of household members. A dietary history was obtained using a food frequency questionnaire.

#### 2.4.2. Exposure Assessment

Self-reported questionnaire information: The baseline questionnaire was used to ascertain household fuel use, including the type of fuels—wood or other biomass materials (cow dung, grass), kerosene, liquid petroleum gas (LPG), or electricity; their frequency and duration of use; the purpose of fuel use—cooking meals, boiling water, and/or heating; and location of cooking—indoor or outdoor. The study population was divided into two exposure groups based on their fuel use as reported by the respondents: a ‘high exposure group’, comprising those who used biomass fuels and kerosene as the primary cooking fuel, and a ‘low exposure group’, comprising those who used electricity or liquefied petroleum gas as the primary cooking fuel. In the subsample of households in which air quality measurements were made, the reported fuel type was used in the preparation of the main lunch meal.

Air quality measurement (AQM): AQMs for PM_2.5_, CO, and carbon dioxide (CO_2_) were conducted in a subsample of 250 randomly selected households (~50% in each of the exposure groups) from the total, three times during the pregnancy (once in each trimester). Measurements were taken in the kitchen during the cooking of the lunch meal, the main meal of the household, using two real-time monitors for a 2 h period. A TSI (TSI Incorporated, 500 Cardigan Road, Shoreview Road, Robbinsdale, MN, USA) DustTrak II monitor (DUSTTRAK™) was used to measure the PM_2.5_ levels. The TSI’s Q-trak monitor was used to measure the CO_2_ and CO levels. Its sensors display real-time, simultaneous CO_2_ and CO concentrations. A zero calibration was performed before installing the instruments at the monitoring locations. A standard protocol was established to install the monitors in the kitchen area. The monitor receiver inlet was kept 145 cm above the floor, 100 cm from the cooking stove, and at least 150 cm away from windows and doors opening outwards. In some instances, slight modifications were made to effectively monitor the available space within the kitchen. The maximum deviation from these standard specifications was less than 10 cm. A standard measuring tape was used to measure distances. The recordings of air quality measurements were extracted daily and entered in a study database.

#### 2.4.3. Outcome Assessment

Anthropometric measurements: At birth, the weight (in grams) and length (in cm) were measured using standard equipment.

The Brazelton Neonatal Behavioural Assessment Scale (BNBAS): Paediatricians trained by a developmental psychologist assessed the neurodevelopment of each child at birth using the BNBAS. The BNBAS scale is a neurobehavioural assessment scale and regarded as the most comprehensive examination of newborn behaviour available. The BNBAS assumes that a newborn infant is both competent and complexly organized and allows doctors to see how a newborn’s discrete behaviours are integrated into coherent patterns of behaviour and development. The examination does not yield a single score but instead assesses a baby’s capabilities across different developmental areas and describes how the baby integrates these areas as he/she deals with their new environment [16]. The BNBAS assesses a newborn’s behavioural repertoire with 28 behavioural items, each scored on a nine-point scale. It also includes an assessment of the infant’s neurological status on 20 items, each scored on a four-point scale [16,17]. Lower scores indicate poor development. The domains assessed by the scale are as follows: habituation (sleep protection), social interactive responses and capabilities, motor system, state organization and regulation, autonomic system, and reflexes. As such, the three outcome variables—birth weight, birth length, and BNBAS scores— were treated as continuous variables in the analyses.

#### 2.4.4. Covariates

Exposure to other environmental pollutants, such as pesticides, insecticides, and lead, were assessed using the questionnaire. Information on the maternal medical history was abstracted from the pregnancy records of the mothers and the CNTH medical records routinely. These included the maternal weight gain during each trimester; maternal haemoglobin; and history of infectious diseases, including sexually transmitted diseases.

### 2.5. Data Analysis

Initially, the data were entered into EPIDATA data bases and imported into SPSS version 26 software and Winpepi software (version 11.65) for further analyses. Descriptive statistics were computed for the continuous and categorical variables and compared by exposure status using a two-sample Student’s T-test for the normally distributed data or chi-square tests for the frequency data. The Mann–Whitney U non-parametric test was used to compare the skewed data for AQM levels by exposure status.

A linear regression model was used to estimate the unadjusted regression coefficients and standard errors for birth outcomes, including birth weight, length, and NBAS scores, in relation to exposure status (high vs. low). A multivariable linear regression model was used to evaluate the association between exposure status (high vs. low) and birth outcomes, adjusting for covariates. Variables were included in the model if the prevalence was >10% in the whole sample, had been decided a priori to be included in the model based on the literature (maternal age, education level, father’s occupation, and pregnancy-related conditions), or were significantly associated with the outcome variable at α = 0.05. All the linear models were adjusted for the father’s and mother’s education, father’s and mother’s age, income, mother’s BMI in first trimester, hypertension in present pregnancy, and gestational diabetes. Correlational analyses using Spearman’s coefficient were used to evaluate the association between the AQMs and birth outcomes.

## 3. Results

**Figure 1:**
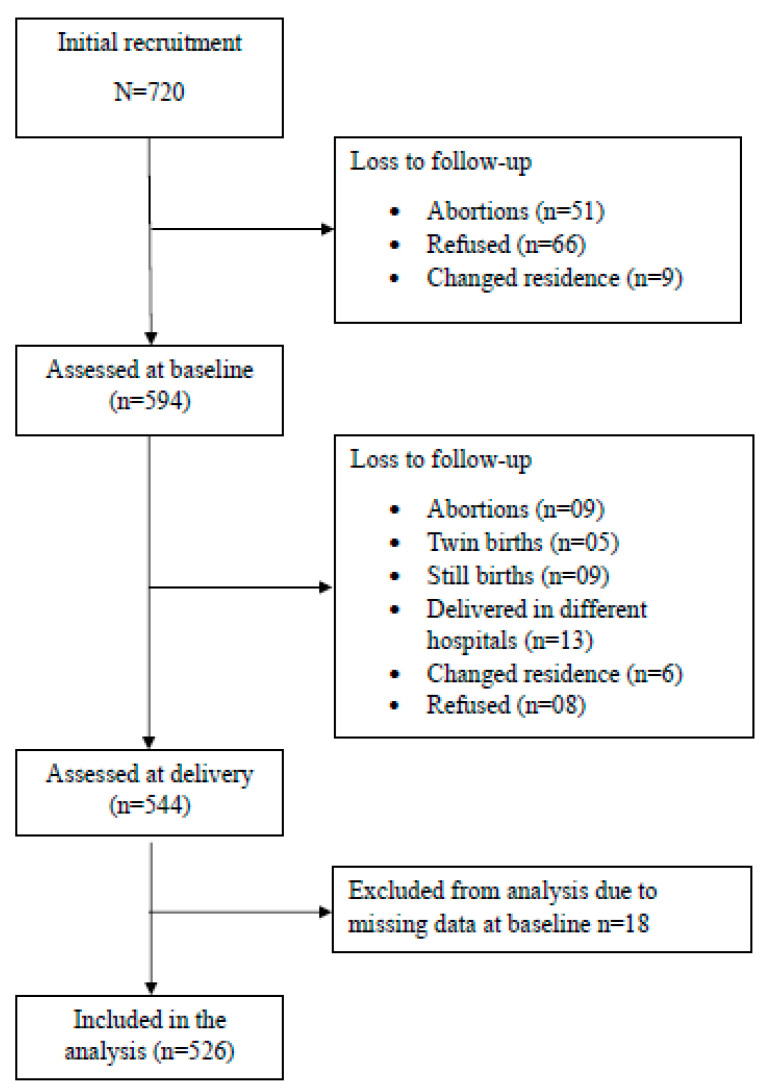
Data flow.

Of the 720 pregnant females who were invited to participate in the study and recruited, 594 were assessed at baseline. Of these 594 females, 526 who had a singleton pregnancy were assessed at delivery. A total of 176 were lost to follow-up due to various reasons (Figure 1). Eighteen pregnant females were excluded from the final analyses due to missing data. Based on the self-reported baseline information on fuel use, 294 (55.9%) mother–child pairs were classified as the high-exposure group and 234 (44.1%) as the low-exposure group.

### Socio-Demographic Characteristics of Families

The comparison of socio-demographic characteristics of families of females who completed the assessment at delivery and at baseline, between the high-exposure group (biomass fuel and kerosene users) and the low-exposure group (LPG and electricity users), are shown in Table 1.

The comparison of the socio-demographic profiles of those who continued till delivery and those who dropped out of the study (not assessed at delivery) is given in Supplementary Table S1. There was no significant difference in the socio-demographic variables between the dropouts, based on the assessment at baseline, and those who continued in the study up to the assessment at delivery. Neither was attrition related to exposure status.

At baseline, there were no differences in the ages of the parents between the high- and low-exposure groups; however, there were differences in the parents’ highest education levels (*p* < 0.001) and income (*p* < 0.001); the parents’ education levels and income were higher in the low-exposure group (Table 1).

The median PM_2.5_ (720 µg/m^3^ vs. 115 µg/m^3^, *p* < 0.01) and CO levels (2000 ppm vs. 1100 ppm, *p* < 0.01) were significantly higher in the high-exposure group. The proportion of family members smoking in the household was higher among the high-exposure group compared to the low-exposure group (63.1% vs. 36.9%, *p* = 0.01) (Table 1). There were no differences in pregnancy-related conditions between the high- and low-exposure groups, except for mother’s body mass index (BMI) in the first trimester (Supplementary Table S2). The maternal BMI was significantly lower among the high-exposure group compared to the low-exposure group (23.4 vs. 21.9, *p* = 0.001).

A total of 177 females were in their first pregnancy, while 338 were in their second or later pregnancy. The parity of 13 females who were assessed at delivery was not recorded. Among the 338 females who were in their second or later pregnancy, 191 were in the high-exposure group and 147 were in the low-exposure group. There was no difference in the past pregnancy-related medical history between the high-exposure and low-exposure females.

There was a significant difference in the mean birthweights of children between the high- and low-exposure groups; the birth weight was lower for the newborns of high-exposure females by 134 g (2889 ± 457 g vs. 3023 ± 480 g, *p* = 0.001) (Table 2). The proportion of children who had a low birthweight (<2500 g) was higher among the high-exposure group compared to the low-exposure group (66.2% vs. 33.8%, *p* = 0.06). There were no differences in birth length (*p* = 0.08), gestational age (*p* = 0.89), pre-term births, or APGAR scores of newborns at 1, 5, and 10 min between the two exposure groups. There was no difference in the outcomes of the BNBAS between the two exposure categories, except for the motor system. The mean score for the motor system was lower for the babies in the high-exposure group compared to the low-exposure group (6.41 vs. 6.55, *p* = 0.038).

The results of the linear regression analyses are provided in Table 3. The birth weights in the high-exposure group were significantly lower than those in the low-exposure group in both the unadjusted and adjusted models (regression coefficient in adjusted model = −106.8; 95% CI: −197.6, −16.0) (Table 3). No association was found between IAP exposure and other birth outcomes, including birth length, gestational age, APGAR scores, or BNBAS scores.

For the subset of the study population with measured indoor PM_2.5_ and CO (n = 303), no significant associations were noted with any of the birth outcomes (Table 4).

## 4. Discussion

Our study reveals that exposure to emissions from the combustion of biomass fuel during pregnancy has a significant impact on the birthweight of the offspring. However, we did not find any association between the use of biomass fuel as the primary cooking fuel and birth length or the BNBAS score at birth, except for the motor system score. The high- and low-exposure groups were comparable in terms of their socio-demographic characteristics, except for income status. The pregnant females were also comparable in both exposure groups in terms of their medical histories and pregnancy-related conditions—hypertension, parity, anaemia, and other pregnancy risks. No notable differences were observed between the pregnant females retained in the study and those excluded from the analyses for multiple reasons.

The mean difference between the birthweight of the neonates in the two groups in this sample was 134 g. After adjusting for other variables, the birthweight of children in the high-exposure group was lower by 107 g compared to that of the low-exposure group. Studies conducted in India have also reported lower birthweights among children of females exposed to IAP during pregnancy. Sreeramareddy et al. (2011) reported a difference of 73 g [18], while Wylie et al. (2014) reported a difference of 112 g [19]. In a more rural setting in Sri Lanka, the risk of low birthweight in children whose mothers were exposed to biomass fumes was 2.74 times higher than those who were not exposed [10]. Similar findings were reported by studies conducted in Bangladesh (OR = 2.6) [5], Ethiopia (aOR 1.5) [7], Zimbabwe [8], and Nigeria [6].

The PM_2.5_ and CO concentrations within households were significantly higher in the high-exposure group. However, there was no correlation between the air contaminant levels and birthweight. A plausible explanation is that the high- and low-exposure categorization was based on the primary fuel used and did not eliminate the possibility of using more than one fuel type. Additionally, the measurement of air contaminant levels was performed only during the preparation of the lunch meal. In a typical Sri Lankan household, the main meal is considered the lunch meal, especially when the female of the household is a housewife. But this may differ depending on the behaviour of the adults. For example, if both parents are working, then breakfast or dinner may be the main meal. In this study, in the households in which the air quality levels were monitored, the lunch meal was prepared by the female in the household. It is also likely that more than one fuel type was used for the cooking of different preparations. For example, oftentimes an electric kettle is used for boiling water and an electric rice cooker is used for cooking rice.

The BNBAS was used to assess newborn behaviour. Apart from birthweight, we detected a significant difference in the BNBAS motor system score between the neonates of the two exposure groups (*p* = 0.038). However, there was no difference between the two groups in terms of the other BNBAS domains. BNBAS is designed to describe the newborn’s responses to his/her extrauterine environment and the contribution of the newborn infant to the development of emerging parent–child relationship on the assumption that the newborn is both competent and complexly organized [17]. The BNBAS is sensitive to monitor newborn infants who have undergone intrauterine stress and have low birthweight, are premature, and small-for-gestational-age [20]. The lack of significant differences in the BNBAS scores between the two groups of infants in this study is likely due to the low percentage of low birthweight and premature births in both groups. The other reasons postulated for the lack of differences in the other indices may also be contributors to the findings from the BNBAS results.

The studies that have investigated the mechanism behind exposure to IAP and adverse birth outcomes suggest that particulate and gaseous air pollution is associated with increased resistance to blood flow in the uterine circulation system. Moreover, the evidence suggests that exposure to air pollution during pregnancy may be associated with oxidative stress, inflammation, and epigenetic alterations in placental tissue and/or maternal cord blood that may lead to outcomes such as suboptimal measures of foetal growth, preterm birth, and stillbirth [21].

Even though the birthweight of newborns in the high-exposure group was significantly lower than that in the low-exposure group, the average birthweight of the neonates in the high-exposure group was within the comparable limits of average birthweight [22]. A possible reason could be the availability of good primary healthcare for all pregnant females in the country, where mothers are individually monitored throughout their pregnancy and interventions are provided to maintain all parameters within normal ranges. Another reason could be different cooking patterns, such as having kitchens outdoors and the availability of improved stoves [23,24] and chimneys to direct emissions outside, which can reduce exposure to pollutants even during biomass combustion. In some houses, the kitchens are located outside the living area, which further reduces exposure to airborne contaminant concentrations during combustion of biomass fuels.

Our study has several strengths that enhance the robustness of our findings. The sample size was sufficient for both exposure groups to detect differences in birthweight by exposure status. The prospective design of the study, comparable baseline characteristics between the groups, the robust and rigorous measurement of birth outcomes, and the detailed medical histories taken before and during pregnancy helped minimize potential confounding. The few who were lost to follow-up after the baseline assessment comprised less than 5% (26/544) of eligible mother–child dyads assessed at baseline. Additionally, our study was set in Sri Lanka, one of the few low–middle income countries with a strong primary healthcare system and maternal–child health indicators comparable to those of high-income countries. Routine prenatal monitoring of and nutritional support for pregnant women further reduced confounding from factors such as nutritional deficiencies and infectious diseases, which are often associated with low birthweight and other adverse outcomes.

### Limitations

We acknowledge some limitations in this study. The outdoor air quality, which is a potential contributor to IAP, was not assessed in this study. It is possible that outdoor pollutants may have affected the indoor air pollutant levels. The indoor air quality measurements were performed at times when traffic congestion was minimal in the area (10:00 h to 12:00 h). None of the houses were located close to an air-polluting industry. Hence, we feel that our results are valid and the association we demonstrate is real.

Moreover, indoor air quality measurements were performed only during the preparation of the mid-day meal. Most of the participants used a combination of different types of fuels. The choice of the primary fuel for cooking also depends on affordability; for example, when liquefied petroleum gas prices increase, many households revert to the use of biomass fuel for cooking. The categorization was conducted based on the primary fuel type used for cooking as reported by the participant; the categorization into the two groups was based on the WHO classification of clean fuels. As adjustments for different fuel types used during the study period were not made, it is likely that a misclassification bias may have occurred, especially in the group classified as users of electricity and LPG.

We did not make any correction for multiple comparisons when analysing the individual BNBAS domain scores; there were no significant differences in the individual domain scores between the two exposure groups except for the motor system score, which was unlikely to have significantly changed the results of this study or their interpretation.

## 5. Conclusions

Maternal exposure to IAP from biomass fuel combustion can negatively impact birthweight. Air contaminant levels were significantly higher in the kitchens of households using biomass fuels for cooking compared to those using electricity or liquefied petroleum gas. Despite the widespread use of biomass fuels and increased IAP exposure in many homes, the presence of a robust primary and antenatal healthcare system, along with a diet rich in green vegetables and seafood, may have mitigated some of the adverse effects of IAP exposure. Further research is needed to assess the long-term impact of IAP on birth outcomes, particularly in vulnerable populations with limited access to healthcare and nutritional resources.

## Figures and Tables

**Table 1:** Comparison of socio-demographic characteristics of pregnant mothers in high- and low-exposure groups who were assessed at baseline and followed up at delivery (n = 526).

Variable	High Exposure N (%)	Low Exposure N (%)	Chi-Square Test (*p*-Value)
Mother’s age (years) (mean (sd))	28.4 (4.7)	28.9 (5.0)	t_524_ = 0.968 (*p* = 0.333)
Spouse’s age (years) (mean (sd))	31.7 (4.7)	32.6 (5.1)	t_519_ = 2.007 (*p* = 0.045)
Mother’s education level			
Grade 10 or less	24 (68.6)	11 (31.4)	$\chi_{2}^{2}=10.662$ (*p* = 0.005)
Up to GCE ordinary level	207 (58.8)	145 (41.2)	
GCE advanced level or graduate	62 (44.6)	77 (55.4)	
Spouse’s education level			
Grade 10 or less	21 (77.8)	06 (22.2)	$\chi_{2}^{2}=13.584$ (*p* < 0.001)
Up to ordinary level	216 (58.1)	156 (41.9)	
Advanced level or graduate	55 (43.7)	71 (56.3)	
Income category (in LKR ^1^) (n = 521)			
<25,000	191 (68.2)	89 (31.8)	$\chi_{1}^{2}=38.644$ (p<0.001)
≥25,000	99 (41.1)	142 (58.9)	
History of family member smoking			
Yes	106 (63.1)	62 (36.9)	$\chi_{1}^{2}=5.466$ (*p* = 0.019)
No	187 (52.2)	171 (47.8)	
PM_2.5_ (µg/m^3^) (median (IQR)) (n = 303)	720	115.5	Mann–Whitney U test (*p* < 0.001)
	(208–1860)	(65.5–256)	
	(n = 168)	(n = 135)	
CO (ppm) (median (IQR)) (n = 258)	2000	1100	Mann–Whitney U test (*p* < 0.001)
	(1200–4066)	(700–1300)	
	(139)	(n = 119)	

^1^ LKR refers to Sri Lankan Rupees (USD 1 ≈ LKR 135) at the time of the study.

**Table 2:** Association between birth outcomes and primary fuel type.

Variable	High Exposure ^1^	Low Exposure ^2^	Statistical Test (*p*-Value)
Birthweight (n; mean (sd) in g) (n = 526)	293; 2889 (457. 3)	233; 3023 (480.5)	t_542_ = 3.268 (*p* = 0.001)
Low birth weight (n = 526)			
Yes (n (%)) No (n (%))	45 (15.3) 248 (84.7)	23 (9.8) 210 (90.2)	$\chi_{1}^{2}=3.384$ (p=0.06)
Birth length (cm) (n; mean (sd))	291; 50.12 (3.15)	231; 50.60 (3.17)	t_520_ = 1.711 (*p* = 0.08)
Gestational age (days) * (n; mean (sd)) (n = 469)	260; 271.29 (10.8)	209; 271.15 (10.7)	t_467_ = −0.134 (*p* = 0.89)
Pre-term birth (n = 45)			
Yes (n, (%))	21 (7.2)	24 (10.3)	Fisher’s exact test (*p* = 1.00)
No (n, (%))	272 (92.8)	209 (89.7)	
APGAR score (n; mean (sd))			
1 min	288; 9.56 (1.03)	229; 9.56 (0.94)	t_515_ = −0.07 (*p* = 0.94)
5 min	287; 9.92 (0.39)	227; 9.95 (0.23)	t_512_ = 0.827 (*p* = 0.409)
10 min	287; 9.96 (0.21)	226; 9.94 (0.62)	t_511_ = −0.778 (*p* = 0.437)
Brazelton Neonatal Behavioural Assessment Scale (BNBAS) scores			
Smiles (n; mean (sd))	138; 0.79 (1.09)	118; 0.85 (1.14)	t_254_ = 0.471 (*p* = 0.638)
Habituation (n; mean (sd))	216; 7.15 (0.94)	172; 7.14 (0.95)	t_386_ = −0.134 (*p* = 0.893)
Social interaction (n; mean (sd))	253; 5.77 (0.85)	195; 5.80 (0.91)	t_446_ = 0.294 (*p* = 0.769)
Motor system (n; mean (sd))	252; 6.41 (0.69)	196; 6.55 (0.64)	t_446_ = 2.077 (*p* = 0.038)
State organization (n; mean (sd))	252; 5.68 (0.97)	196; 5.66 (0.95)	t_446_ = −0.156 (*p* = 0.876)
State regulation (n; mean (sd))	252; 6.27 (0.96)	196; 6.29 (1.01)	t_446_ = 0.240 (*p* = 0.810)
Autonomic system (n; mean (sd))	252; 5.19 (0.97)	196; 5.09 (0.94)	t_446_ = −1.048 (*p* = 0.295)
Supplement items (n; mean (sd))	252; 6.35 (0.77)	196; 6.30 (0.72)	t_446_ = −0.566 (*p* = 0.572)
Reflexes (n, mean (sd))	252; 1.64 (0.16)	196; 1.65 (0.15)	t_446_ = 0.740 (*p* = 0.460)

^1^ Low = electricity and liquid petroleum gas (LPG) as primary fuels; ^2^ High exposure = use of biomass wood and kerosene as primary fuels. * Gestational age is the number of days from the first day of the last menstrual period until delivery.

**Table 3:** Birth outcomes at delivery.

Birth Outcome	Exposure ^1^	Unadjusted			Adjusted ^2^		
		Regression Coefficient (β)	*p*-Value	95% CI of β	Regression Coefficient	*p*-Value	95% CI of β
Birth weight (g)	Low	0.000					
	High	−134.1	0.001	−214.8–(−53.5)	−106.8	0.003	−197.6–(−16.0)
Birth length (cm)	Low	0.000			0.000		
	High	−0.477	0.08	−1.024–0.071	−0.474	0.203	−1.205–0.257
Gestational age (days) *	Low	0.000			0.000		
	High	0.135	0.89	−1.843–2.113	−1.459	0.255	−3.975–1.056
APGAR score (5 min)	Low	0.000			0.000		
	High	−0.025	0.40	−0.083–0.034	−0.055	0.185	−0.136–0.026
Brazelton Neonatal Behavioural Assessment Scale (BNABS)	Low	0.000			0.000		
	High	−0.133	0.038	−0.259–(−0.007)	−0.130	0.075	−0.272–0.013

^1^ Low = electricity and liquid petroleum (LPG) as primary fuels; high exposure = use of biomass wood and kerosene as primary fuels. ^2^ Models adjusted for hypertension in present pregnancy, gestational diabetes, father’s and mother’s education, father’s and mother’s ages, mother’s BMI in first trimester, and income. * Gestational age is the number of days from the first day of the last menstrual period until delivery.

**Table 4:** Spearman correlation coefficients between birth outcomes and PM_2.5_ and CO.

Indoor Air Pollutant	Birth Weight	Birth Length	Gestational Age	APGAR Score	Neonatal Behavioural Assessment Scale (NBAS)
Particulate matter (PM_2.5_)	r = −0.009 (n = 303) (*p* = 0.878)	r = −0.033 (n = 302) (*p* = 0.573)	r = 0.052 (n = 273) (*p* = 0.388)	r = 0.075 (n = 295) (*p* = 0.201)	r = 0.011 (n = 258) (*p* = 0.858)
Carbon monoxide (CO)	r = −0.007 (n = 258) (*p* = 0.917)	r = −0.064 (n = 257) (*p* = 0.305)	r = 0.03 (n = 230) (*p* = 0.651)	r = 0.02 (n = 251) (*p* = 0.653)	r = −0.044 (n = 219) (*p* = 0.519)

## Data Availability

The datasets generated and/or analysed during the current study are not publicly available due to the request to do so not being approved by the Ethics Review Committee, but they are available from the corresponding author on reasonable request. The contact details of the Ethics Review Committee are as follows: Ethics Review Committee, Faculty of Medicine, University of Kelaniya, P.O. Box 6, Thalagolla Road, Ragama 11010, Sri Lanka (Tel.: +94112961267: Email—ercmed@kln.ac.lk).

## Supplementary Materials

The following supporting information can be downloaded at: https://www.mdpi.com/article/10.3390/ijerph22091336/s1, Table S1. Comparison of socio-demographic profiles of participants assessed at baseline who had dropped out before delivery. Table S2. History of current pregnancy.

## Abbreviations

APGAR	Appearance, Pulse, Grimace, Activity, and Respiration
BMI	Body Mass Index
BNBAS	Brazelton Neonatal Behavioural Assessment Scale
CHDR	Child Health Development Record
CNTH	Colombo North Teaching Hospital
CO	Carbon Monoxide
CO_2_	Carbon Monoxide
DCS	Department of Census and Statistics
HAP	Household Air Pollution
IAP	Indoor Air Pollution
IAQ	Indoor Air Quality
IQR	Interquartile range
LPG	Liquified Petroleum Gas
MCH	Maternal and Child Health
MOH	Medical Officer of Health
PHI	Public Health Inspector
PHM	Public Health Midwife
PM	Particulate Matter
RA	Research Assistant
UKe	University of Kelaniya
