# Supplementary material for: Effect of Biomass Fuel Use on Neonatal Outcomes: A Cohort Study of Pregnant Females
Source: Int J Environ Res Public Health. 2025 Aug 27;22(9):1336. doi: 10.3390/ijerph22091336 (PMC12469654; doi:10.3390/ijerph22091336)
Supplement: Supplementary file 1 [file ijerph-22-01336-s001.zip › ijerph-3753826-supplementary.pdf]

Table S1. Comparison of socio-demographic profiles of participants and drop-outs

Variable at baseline (T0)	Category	Total Sample at baseline (T0)	Continued to delivery (T1)	Dropped out	Statistical test[1] (p-value)
Mother's age (mean (sd))		28.71 (5.04)	28.65 (4.90)	29.18 (6.06)	t_{79}=0.549 (p=0.589)
Father's age (mean (sd))		32.29 (5.15)	32.16 (4.97)	33.33 (6.36)	t_{78}=1.25 (p= 0.212)
Mother's age category (n=594) (n (%))	15- 29 years	328 (55.2)	296 (56.1)	32 (48.5)	$\chi^2_1 = 1.362$ (p = 0.243)
	30-45 years	266 (44.8)	232 (43.9)	34 (51.5)	
Mother's highest education category (n=593) (n (%))	Grade 10 or less	39 (6.6)	35 (6.6)	04 (6.2)	Fisher's exact test (p=0.927)
	Up to ordinary level	396 (66.8)	354 (67.0)	42 (64.6)	
	Advanced level or Graduate	158 (26.6)	139 (26.3)	19 (29.2)	
Father's highest education category (n (%))	Grade 10 or less	31 (5.2)	27 (5.1)	04 (6.2)	Fisher's exact test (p = 0.648)
	Up to ordinary level	417 (70.4)	374 (71.0)	43 (66.2)	
	Advanced level or Graduate	144 (24.3)	126 (23.9)	18 (27.7)	
Father's occupation (3 categories) (n (%))	Managerial, Professional, and Technical Support	174 (35.0)	157 (34.9)	17 (36.2)	$\chi^2_2 = 5.338$ (p = 0.070)
	Armed Forces and Agriculture related	203 (40.8)	190 (42.2)	13 (27.7)	
	Elementary occupations	120 (24.1)	103 (22.9)	17 (36.2)	
Income category (based on Sri Lankan Rupee, 1 USD ≈ 135 LKR	< 25000	317 (54.2)	281 (53.7)	36 (58.1)	χ^2_1 =0.420 (0.517)
	$\geq$ 25000	268 (45.8)	242 (46.3)	26 (41.9)	

Variable at baseline (T0)	Category	Total Sample at baseline (T0)	Continued to delivery (T1)	Dropped out	Statistical test[1] (p-value)
at the time of the baseline survey) (n=589) (N (%))					
Someone in the family smoking (n (%))	Yes	185 (31.1)	168 (31.8)	17 (25.8)	$\chi_1^2 = 1.005$ (p=0.316)
	No	409 (68.9)	360 (68.2)	49 (74.2)	
Primary fuel used (n (%))	LPG or Electricity (low exposure group)	267 (44.9)	234 (44.3)	33 (50.0)	$\chi_1^2 = 0.765$ (p=0.432)
	Wood or Kerosene (high exposure group)	327 (55.1)	294 (55.7)	33 (50.0)	
Particulate matter ($PM_{2.5}$) (median (IQR))		259 (87.7 -1100) (n=288)	255.0 (90.0-1071.0) (n=259)	319.0 (63.5 - 1650.0) (n=29)	Mann Whitney test (p=0.933)
Carbon Monoxide (CO) (median (IQR))		1300 (900.0-2700.0) (n=288)	1300 (900.00-2800.0) (n=259)	1300 (966.97-2433.3) (n=29)	Mann Whitney test (p=0.832)

[1] Comparison between those who continued till delivery and those who dropped out.

Table S2. History of current pregnancy

Variable	Category	**High exposure**	**Low exposure**	**Statistical test (p-value)**
Parity[1] (N=515) (n (%))	Primi-para	96 (54.2)	81 (45.8)	$\chi_2^2 = 1.100$ (p = 0.577)
	Para 2	118 (54.6)	98 (45.4)	
	Para 3 or more	73 (59.8)	49 (40.2)	
Mother's BMI in first trimester (mean (sd) in Kg/height in m^2) (N=593)		21.9 (4.3)	23.4 (4.4)	t_{430}=3.61 (p=0.001)
Other pregnancy risks (N=526) (n (%))	Yes	05 (62.5)	03 (37.5)	Fisher's exact test (p=1.000)
	No	288 (55.6)	230 (44.8)	
Gestational hypertension (N=526) (n (%))	Yes	14 (56.0)	11 (44.0)	$\chi_1^2 = 0.001$ (p=0.976)
	No	279 (55.7)	222 (44.3)	
Gestational diabetes (N=526) (n (%))	Yes	10 (55.6)	08 (44.4)	$\chi_1^2 = 0.000$ (p=0.990)
	No	283 (55.7)	225 (44.3)	
Anaemia (N=526) (n (%))	Yes	03 (60.0)	02 (40.0)	Fisher's Exact test (p=1.000)
	No	290 (55.7)	231 (44.3)	
Abnormal pregnancy (N=526) (n (%))	Yes	(0)	03 (100.0)	Fisher's exact test (p=0.086)
	No	293 (56.0)	230 (44.0)	

[1] There were missing values.
